# Left Ventricular Thrombosis in Ischemic and Non-Ischemic Cardiomyopathies: Focus on Evidence-Based Treatment

**DOI:** 10.3390/jcm14051615

**Published:** 2025-02-27

**Authors:** Filippo Catalani, Simone Sarzilla, Massimiliano Will, Giovanni Pedrazzini, Andrea Demarchi

**Affiliations:** 1 Department of Internal Medicine, Regional Hospital of Bellinzona e Valli, Ente Ospedaliero Cantonale, 6500 Bellinzona, Switzerland; filippo.catalani@eoc.ch (F.C.); simone.sarzilla@eoc.ch (S.S.); massimiliano.will@eoc.ch (M.W.); 2 General Internal Medicine and Thrombotic and Hemorrhagic Diseases Unit, Department of Internal Medicine, University of Padova, 35128 Padua, Italy; 3 Division of Cardiology, Cardiocentro Ticino Institute, Ente Ospedaliero Cantonale, 6900 Lugano, Switzerland; giovanni.pedrazzini@eoc.ch; 4 Faculty of Biomedical Science, Università della Svizzera Italiana, 6900 Lugano, Switzerland; 5 Division of Cardiology, Cardiocentro Ticino institute, Ente Ospedaliero Cantonale, 6500 Bellinzona, Switzerland

**Keywords:** left ventricular thrombosis, LVT, thrombosis, anticoagulation, VKA, DOAC, acute coronary syndrome, ischemic cardiopathy, cardiomyopathy

## Abstract

Left ventricular thrombosis (LVT) is one of the most feared complications of both ischemic and non-ischemic cardiopathy, and despite its incidence having decreased over the years (mostly due to novel reperfusion therapies in acute coronary syndromes), it is still not negligible. If transthoracic echocardiography, possibly with the adjunction of echo contrast, represents the cornerstone in LVT diagnosis, sometimes it is found to be nonconclusive and advanced cardiovascular imaging, namely cardiac magnetic resonance, needs to be performed to fully exclude intraventricular masses or to better characterize them. Vitamin K antagonists always represented the anticoagulant of choice for the treatment of LVT; however, the recent spread of direct oral anticoagulants (DOACs) pushed clinicians to adopt them also in this setting despite the absence of robust evidence in their favor. If the optimal duration of anticoagulation for the treatment of LVT in non-ischemic cardiopathy is still a matter of debate, an initial treatment of 3–6 months seems to be reasonable in the setting of ischemic cardiopathy, with possible extension according to the follow-up findings. High-quality randomized studies are strongly needed to evaluate the potential role of prophylactic anticoagulation in high-risk patients and provide conclusive evidence for the use of DOACs in LVT treatment.

## 1. Introduction

Left ventricular thrombosis (LVT) is historically related to ischemic cardiomyopathy (ICM), which is usually detected during the evaluation in the acute phase of a myocardial infarction (MI) or during its follow-up, with a prevalence of 4.7–26%. The data on the presence of LVT in non-ischemic cardiomyopathies (NICMs) are limited and scattered, and no complete comparison has been made in terms of epidemiology, pathophysiology, and therapy between these two different entities. Considering the non-negligible prevalence in the latter groups, with dilated cardiomyopathy (DCM) being the major responsible factor, we attempted to fill this gap. We sorted out why, in different ways, all these pathologies could represent -at a certain stage- the perfect environment for the formation of the thrombus, characterized by endothelial damage, blood stasis, and hypercoagulability, namely Virchow’s triad. Transthoracic echocardiography (TTE) is still the primary diagnostic tool for LVT in general, thanks to its wide use as the first imaging evaluation tool and the use of scores such as apical wall motion scoring can overcome its limitations due to the limited acoustic windows. Nonetheless, cardiac magnetic resonance (CMR) remains the gold standard, showing superiority in both the diagnosis and the monitoring of the evolution of LVT.

Regarding the therapeutic strategy for LVT in ICM, the latest guidelines for the treatment of acute coronary syndromes (ACS) by the European Society of Cardiology (ESC) and the American Heart Association (AHA) classify direct oral anticoagulants (DOACs) with the same level of evidence as vitamin K antagonists (VKAs) and suggest an individualized approach for both prevention strategies and treatment duration. In the setting of LVT and NICM, only few data are available regarding the choice of the agent, the duration of treatment and the strategy of prevention. Through the following sections, we will illustrate the current literature and future perspectives on the topic.

## 2. Epidemiology

The overall prevalence of LVT in the general population is low. A retrospective review that analyzed around 85,000 medical images of two-dimensional TTE, CMR, and coronary CT angiography (CTA) showed that the incidence of LVT was 7 per 10,000 patients. Patients with LVT had ICM in 80% of cases, while the remaining were affected by NICM: 8.15% had DCM, and 4.8% had stress-induced Takotsubo cardiomyopathy (TTC) [1].

A nationwide study including almost 2700 patients from the JROAD-DPC (Japanese Registry of All Cardiac and Vascular Diseases Diagnosis Procedure Combination) showed a similar proportion between ICM and NICM, confirming in the latter group that DCM, TTC and hypertrophic cardiomyopathy (HCM) were the major responsible for this complication [2].

### 2.1. Ischemic Cardiopathy

As the data show, left ventricular (LV) thrombus in the past decades was mostly associated as a complication of ICM, in particular anterior ST segment elevation MI (STEMI), with an incidence that varies widely from the different reports considered. We must distinguish between the era when acute MI was treated with systemic thrombolysis, roughly until the mid-1980, when the incidence was around 19.4% to 39.6%, and the current era, which is characterized by the widespread use of percutaneous coronary intervention (PCI) that also helped to drastically reduce the percentage of thrombotic complications, with an incidence of LVT formation that varies between 4.7% and 26% [3]. In addition, LVT incidence has a strict relationship with the degree of myocardial damage and the involvement of the LV apex: indeed, in anterior MI compared to non-anterior MI, the chances of developing a thrombotic formation are much higher (11.5% versus 2.5%) [4].

A 2018 meta-analysis by Bulluck et al. [5] showed a predominant association between LVT and anterior STEMI, with 96% of the thrombi occurring in this infarct location. The analysis included six studies comprising 2339 patients with STEMI, consistently demonstrating a significantly higher prevalence of LVT in anterior infarctions. No precise data were available regarding the incidence of LVT in non-anterior infarctions. More recently, a 2024 meta-analysis by Sacoransky et al. [6] confirmed this trend by analyzing 18 studies and reporting an overall LVT prevalence of 5.6%. Among the 16 studies providing data on infarct location, between 81% and 100% of LVTs were associated with anterior STEMI. Additionally, in cases of LVT not resulting from anterior MI, the right coronary artery was identified as the primary culprit vessel, accounting for 2% to 3% of the total cases.

Fewer data are available on the causal relationship between non-ST segment elevation MI(NSTEMI) and the formation of LVT: a retrospective study analyzed a total of 1,379,000 hospitalized patients, showing an incidence of 0.13% of LVT, which was increased in those who received a delayed PCI (>15 days from the onset of symptoms) and in those who developed a heart failure while being hospitalized [7].

### 2.2. Non-Ischemic Cardiomyopathy—Familial and Non-Dilated Cardiomiopathy

Regarding NICMs, the availability of data is limited. A retrospective cohort study analyzed 1267 patients diagnosed with DCM, of whom 89 (7.08%) had LVT (78/89 cases, 87.64%), while biventricular thrombus was rare (4/89 cases, 4.49%) [8]. Unfortunately, we found no data to compare the differences in the incidence of LVT between familial and non-familial forms of DCM.

### 2.3. Non-Ischemic Cardiomyopathy—Others

The most recent reviews, which have encountered 600 cases with documented TTC, show that LVT was detected in about 2.5% of the patients [9]. Most of the cases presented a classical pattern of stressed-induced cardiomyopathy, but formation of thrombus has been anecdotally described in other variants, such as the mid-ventricular variant [10]. In 2017, Rowin et al. reported that out of 1940 patients with HCM, 93 showed an apical aneurysm and 18 had a thrombus in the aneurysm [11], showing an incidence of LVT in HCM of 0.92%. Regarding other cardiomyopathies, in a series published by Feng et al. on cardiac amyloidosis, intracardiac thrombosis was found in 42 of 159 patients, but only 12% of them were located in the LV [12]. Thromboembolic events (stroke and any clinically detectable embolism, such as coronary embolism or LVT) have a median incidence of 2.7% in left ventricular non-compaction (LVNC) in adult and paediatric patients, according to a recent meta-analysis [13]. The incidence is higher in the adult population, with a prevalence of thromboembolic events of 6.2% and, in particular, LVT was found to complicate the follow-up in 5 out of 105 patients who were regularly evaluated with serial TTE [14].

Eventually, according to a register of patients with severe depression of left ventricular ejection fraction (LVEF), namely < 30%, treated with Mitraclip, independently from the etiology of systolic dysfunction, LVT showed a prevalence of 1.1% during a 6-month echocardiographic screening [15]. In some autoimmune diseases, such as Behçet’s disease [16], uncommon cardiac involvement can lead to LVT formation in 1.5% of cases [17] as well as in the hypereosinophilic syndrome, in which -after an initial phase of eosinophil infiltration- the denuded myocardium reacts with a thrombotic-necrotic process in both ventricles, particularly in the ventricular apices [18].

## 3. Pathophysiology and Risk Factors

The pathophysiology of the formation of LVTs and, in general, of thrombotic diseases affecting either the venous or the arterial system is based on the principles of Virchow’s triad: endothelial injury or dysfunction, blood stasis, and hypercoagulability. However, not all of the above heart diseases produce in the same way the environment for the development of LVT.

In ICM, the LV mural thrombus that is formed in the early phase of MI is composed of fibrin, red blood cells, and platelets [19] and its formation is more common after a STEMI compared to NSTEMI, given a major extension of the damage across the myocardial wall and the LV function reduction.

### 3.1. Ischemic Cardiomyopathy

Following a MI, both the structure and the function of endothelial cells are impaired due to the ischemic damage. The loss of integrity of the endothelial layer determines per se an increase in vascular permeability (such as during inflammation), shifting fluids, albumin, and molecules with similar molecular weight, including antithrombin and protein C, from the intravascular compartment into the extravascular space, reducing the amount of natural anticoagulants while at the same time increasing blood viscosity [20].

In addition, dysfunctional endothelial cells contribute to the pathogenesis of thrombosis by altering the expression of pro- and antithrombotic factors: an increase in proinflammatory cytokines, such as TNFα and interleukin-1, upregulates the production of tissue factor (TF) and Von Willebrand factor while attenuating the expression of thrombomodulin, NO, and PGI2 [21,22]. TF has a major role in thrombus formation, by promoting thrombin generation through the direct activation of factor X by the TF-factor VIIa complex [23]. Hypoxia was also found to promote endothelial release of Von Willebrand factor and platelet binding [24]. Of note, the endothelial expression of TF in vivo is controversial: indeed, most of the studies evaluating TF expression in activated endothelial cells have been performed in cultured cells [25].

Inflammatory changes on the endocardial surface may induce platelet deposition and fibrin net formation through interactions with proinflammatory cytokines, such as fibrinogen, IL-6, and C-reactive protein (CRP) [26].

In cultured monocytes, IL-6 favors a thrombotic process either directly by increasing TF expression [27] or indirectly by inducing the hepatic synthesis of CRP, which increases TF procoagulant activity [28]. CRP itself contributes to local inflammation [29] and thrombus formation because it activates the complement system and coagulation cascades, specifically on the endocardial surface of myocardiocytes by binding to phosphatidylcholine [30], a protein formed due to the combination of type IIA secretory phospholipase A2 and lysophosphatidylcholine during the flip-flop phenomenon of the damaged membrane of cardiomyocytes [31].

Given that the more extensive the myocardial necrosis, the stronger the release of inflammatory mediators, several studies have demonstrated that admission serum CRP [32] and fibrinogen levels are independent predictors of early LV thrombus formation complicating a ACS-STEMI [33].

Eventually, several studies [34,35,36] have shown that strong predictors of LVT formation after MI are both the reduction of LVEF [37], thus emphasizing the importance of blood stasis due to regional LV impairment and the presence of an apical aneurism due to a large myocardial scar [38] in the context of a left anterior descending (LAD) infarct-related artery. A retrospective study by Ung Lim Choi et al. found that patients with LV apical thrombus after anterior MI showed a higher mitral E velocity over the mitral annular E′ velocity ratio and a significantly higher incidence of grade 2 and 3 diastolic dysfunction [39]. In addition, abnormal flow patterns caused by a regional LV dysfunction are closely associated with LV thrombus formation compared with normal flow patterns [40,41].

### 3.2. Dilated Cardiomyopathy

In NICMs, thrombosis is always favored by the presence of elements of the Virchow’s triad. Patients with DCM have a higher prevalence of thrombus, which can occur in the setting of atrial fibrillation or independently from other risk factors of thrombosis, such as in patients with sinus rhythm or in children with no other pathologies [42]. Elevated D-dimer levels and reduced LVEF were independently associated with an increased risk LVT, whereas severe mitral regurgitation could decrease the incidence of LVT [43].

Several studies [44,45,46] have demonstrated using different methods that DCM presents endothelial dysfunction, the degree of which is proportional to the severity of LV dilatation. In general, DCM fibrotic changes, characterized by the transition to the mesenchymal phenotype, lead to an altered expression of the molecular array and transformations in the equilibrium between the pro- and anticoagulant status.

Despite genetic disorders account for only about 30% of DCM, in these forms genetic mutations modify sarcomeric, structural and nuclear membrane proteins [47]: for example, a mutation in the integrin-linked kinase gene or in the laminin-a4 gene determines an alteration of the endothelial wall, leading to its thinning and rupture in animal models [48]. All these mechanisms macroscopically lead to LV remodelling, dilatation, and impairment of contractile function, producing hemodynamic changes that facilitate blood stasis and further activation of the coagulation system, as seen by the significant increase in plasma levels of D-dimers, fibrinopeptide A, and thrombin-antithrombin III complex [49].

### 3.3. Hypertrophic Cardiomyopathy

HCM also poses a risk for thrombus formation in both the “classic” HCM phenotype, defined as the phase of hypertrophic myocardium with a hyperdynamic LV (EF >65%), and the end-stage hypokinetic-dilated phenotype, when the volume of LV increases, and spherical remodeling occurs [50,51].

Flow reduction, turbulent flow patterns, and stagnation can occur in cases of left ventricular outflow obstruction, especially in patients with dynamic obstruction and those with impairment of ventricular systolic function due to regional LV necrosis or apical ballooning, which reduces blood suction from the left atrium [52,53]. LV apical aneurysm, as a hypo/akinetic area, confirmed its role as a pivotal risk factor, significantly increasing the risk of thromboembolic events compared to the HCM population without an LV aneurysm (pooled OR: 6.30; 95%CI: 1.52-26.19;I^2^: 66%) [54].

The hypercoagulable state in HCM is the result of the effects of local and systemic inflammatory states: several studies [55,56,57,58] documented the elevation of circulating inflammatory cytokines/chemokines (TNF-α, IL-6) and increased counts of peripheral white blood cells(i.e., neutrophils, and monocytes). Sustained low-grade myocardial inflammation alters the environment, from coagulation factors to circulating cells, and triggers the invasion of inflammatory cells and fibroblasts, ultimately resulting in myocardial fibrosis [59]. In patients with HCM, both fibrinopeptide A and thrombin-antithrombin III complex levels are elevated [49]. Platelets show morphometric and chemical differences: the mean platelet volume is significantly higher (9.1 ± 0.3 vs. 7.9 ± 0.3 fL, *p* = 0.01) and even higher in patients with hypertrophic obstructive cardiomyopathy compared to those with hypertrophic nonobstructive cardiomyopathy [60], the phosphorus concentration is lower, and the permeability to cations is increased [61]. Moreover, myocardial inflammation enhances platelet activity: the expression of p-selectin, a platelet surface protein, and thrombospondin, a major component of platelet α-granules, is significantly correlated with the number of CD3+ T cells and endothelial/interstitial activation [62].

The presence of extensive late gadolinium enhancement (LGE) on contrast-enhanced CMR is an independent predictor of thromboembolic events [63], reflecting myocardial tissue modifications and a proinflammatory environment in the context of myocardial scarring.

### 3.4. Cardiac Amyloidosis

Cardiac amyloidosis (CA), among infiltrative cardiomyopathy, produces substantial alterations of the thromboembolic profile risk: while, during the first phase, the atrial myopathy favors thrombus formation mainly in the left atrial appendage, the progression of the disease can extend to the ventricular chamber leading to its dilatation and increasing the risk of LVT [64]. While studies disagree on which type of CA determines the higher risk of thrombosis, the presence of atrial fibrillation, poor LV diastolic function, RV wall thickness on TTE, and higher heart rate were found to be independent predictors of thromboembolism in CA [64,65].

The deposition of misfolded proteins between myocytes compromises diastolic function through cardiotoxic mechanisms, namely increasing reactive oxygen species (which alters intracellular calcium handling), impairing lysosomal function and autophagic activity and modifying mitochondrial membrane potential [66,67,68]. Reduced myocardial compliance, increased stiffness, and increased ventricular filling pressure create the hemodynamic conditions for stasis that favors LV thrombus development [69], especially in end-stage disease, when a dilatative evolution is frequently observed [65].

Amyloid fibrin deposits in endothelial cells of small arteries, leading to the proliferation of the medial components and microcirculation impairment, and heart cavities, creating parietal injury and valvular amyloid deposits [70,71]. Transthyretin, light chains, and the acute phase proteins in amyloid A amyloidosis, directly affects the coagulation cascade during coagulation initiation and fibrinolysis regulation [65]: in patients with AL amyloidosis, impairment of the thrombin-antithrombin pathway (lower antithrombin III activity, reduced heparin binding) [72], nephrotic syndrome leading to glomerular loss of natural anticoagulant factors (antithrombin III, protein S), and increased synthesis of procoagulant factors (factor V, VII, fibrinogen) [73] play a pathogenic role in the hypercoagulable state reported in CA.

### 3.5. Left Ventricular Non-Compaction Cardiomyopathy

LVNC has the potential to cause embolic events, especially in the case of LVEF reduction (<40%) [13], independently of the prevalence of atrial fibrillation, secondary to myocardial injury, hypercoagulability due to the overexpression of factor V and endothelin-1, and slower blood flow within the prominent myocardial trabeculation and deep intertrabecular recesses: although there is no conclusive evidence to support this hypothesis [74].

## 4. Diagnosis

In clinical practice, most LVTs are diagnosed incidentally during an echocardiographic evaluation after an MI, while some others are found while looking for an embolic disease after a stroke or other thromboembolic complications: in any case, accurate diagnosis of LVT is crucial for timely intervention and management, and various diagnostic modalities are employed, each offering unique benefits and limitations.

TTE is the first diagnostic tool in the evaluation of patients with ICM to assess LV function and screen for mechanical complications. It is readily available, relatively cheap, and safe, but it is operator-dependent, and there are patients (i.e., obese patients and those with lung emphysema) with an impaired acoustic window that could limit the exam itself.

**Figure 1:**
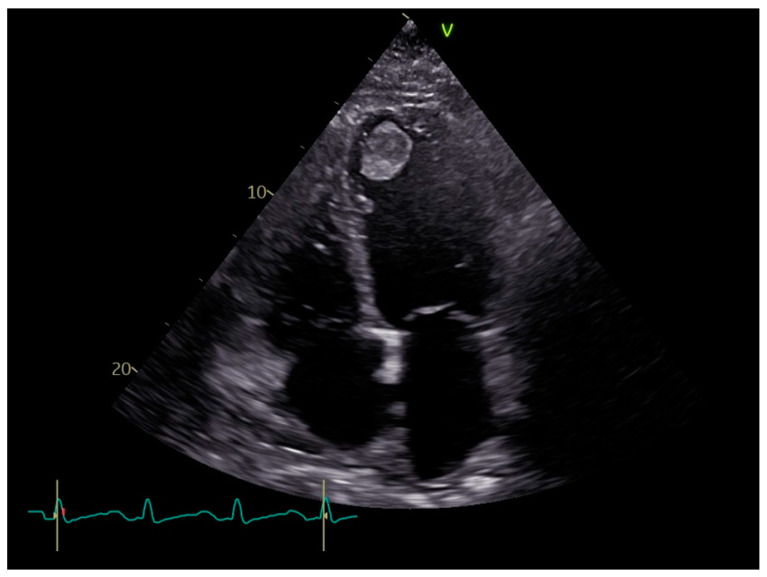
LV apical thrombus detected with TTE, apical 4 chamber view.

LV thrombus is identified as an echo-dense mass in the LV with well-defined margins that are distinct from the endocardium, as shown in Figure 1. It must be observed in at least two views and throughout the whole cardiac cycle, associated within an area with significant LV regional or global wall motion abnormalities [75]. When detected, it could be classified by its shape as mural, a thrombus with a free concave margin that follows the curvature of the LV wall or pedunculated if it is projected in the LV cavity. It is not known which combination of factors promotes the formation of one specific type of thrombus. Pedunculated LV thrombi have a higher embolic potential than the mural ones: in a cohort of 57 patients with pedunculated LVT, 57.8% developed distal emboli at different sites, most commonly brain and the lower limb [76].

Despite recent refinements in the technique, like harmonic imaging, the only anatomical detection of LVT can not guarantee high levels of sensitivity, which varies between 21% and 35% according to studies [77,78] with post-MI patients.

A promising approach to reliably stratify high-risk patients could be adding apical wall motion scoring on routine non-contrast TTE, which evaluates the severity of wall motion abnormalities and could save from visualizing the LVT directly, going beyond the limits of poor image quality or small LVT.

As shown by Weinsaft et al., a high apical wall motion score on TTE (≥5 without contrast, ≥7 with contrast) yielded excellent performance in relation to DE-CMR-evidenced thrombus [36], bringing the sensitivity to approximately 100% and a negative predictive value of 100% for detecting LV thrombi, suggesting which patients could benefit from further imaging with CMR while avoiding unnecessary additional testing [79].

TTE can be useful for monitoring LVT resolution, which has a median period of 3 days, and in some cases, the thrombus can persist for up to 6–12 months [80]. TTE can also be used to screen high-risk patients (apical wall motion impairment, ventricular dysfunction) for further investigation, such as CMR [36]. Unfortunately, mural thrombus and small thrombus are still non-optimally detected by TTE.

The addition of intravenous echo contrast can be used in TTE to increase its diagnostic power: studies have shown an improvement in terms of sensitivity, estimated around 61–64% [36,81], but a recent meta-analysis concluded that in post-MI patients, contrast TTE did not significantly improve sensitivity for LVT compared to non-contrast TTE [82]. Moreover, it is worth mentioning that the use of contrast-echo with Sonovue is contraindicated in patients with recent ACS or with clinically unstable ischemic heart disease.

Transesophageal echocardiography (TEE) has a limited role in the detection of LVT, since cardiac apex is the farthest point from the transducer, and it is usually foreshortened and not well visualized. In a recent meta-analysis of patients post-MI comparing TEE and CMR, the pooled sensitivity and specificity were 58% and 98%, respectively [56]. However, access to TEE is limited by its moderately invasive nature and is contraindicated in patients with significant esophageal disease [83].

**Figure 2:**
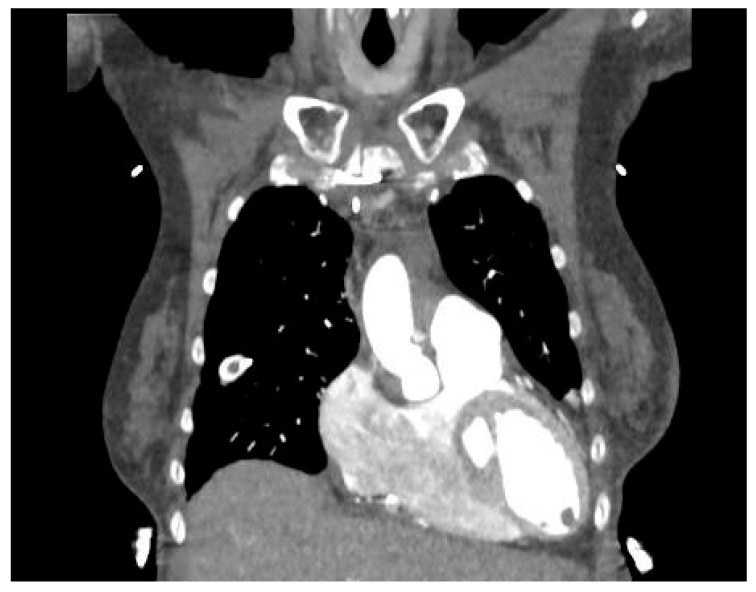
CT with contrast showing a left ventricular apical thrombus.

Contrast-enhanced cardiac computed tomography (CCT) identifies masses with high precision, but tissue characterization is limited, and the appearance of neoplasms and thrombi is similar [84]; that is the reason why LVT can usually be identified as a cavity-filling defect, as shown in Figure 2. A new technology based on spectral tomography dual-substance separation (iodine vs. blood-based) has been proposed to go beyond the suboptimal tissue characterization that arterial and delayed phase contrast can offer [85]. Bittencourt et al. used a threshold of 65 HU to differentiate LVT from the myocardial wall, reaching sensitivity, specificity, and positive and negative predictive values of 94%, 97%, 94% and 97%, respectively [86]. There are few studies comparing different modalities with CCT: a small study in post-MI patients showed an accuracy of 87% [87] in the diagnosis of LVT, while specificity was 100%. A few case reports in NICM also showed the proficient use of CCT in detecting LVT that was initially missed on echocardiography [88,89]. Thus, the risks of ionizing radiation exposure and contrast-related nephropathy limit the broad application of this imaging modality.

In contrast to CCT, which is prone to motion artifacts due to the cardiac cycle, photon-counting detector CT (PCCT) reduces these artifacts while also provides better spatial resolution and enhanced contrast compared with conventional energy-integrating detectors [90]. So far, this advantage has been particularly recognized in the evaluation of coronary artery stenosis [91,92]. Although randomized studies and meta-analyses are currently lacking, case reports have demonstrated the potential of this technique in detecting ventricular thrombi with high accuracy, particularly in the context of small mural thrombi, which may be difficult to distinguish from myocardial tissue [93,94]. Further studies are needed to validate its clinical applicability.

Contrast ventriculography has poor sensitivity (30%) due to the low spatial resolution but provides good specificity (85–90%) [95]. It has important limitations in temporal terms because it is usually performed in the acute phase of MI when the formation of thrombus is uncommon, in repeatability, not being practical for thrombus resolution monitoring, and in safety because it brings a small risk of systemic embolization during catheter manipulation.

Currently, CMR is considered the gold standard technique for LVT detection by gadolinium perfusion and late gadolinium enhancement, owing to its high spatial and temporal resolution and high soft-tissue contrast. It can determine the presence of LVT by recognizing avascular tissue characteristics, which is the feature that distinguishes it from the myocardium, despite the possibility of neovascularization at some stage of its development, described as a sign of mural thrombosis [96]. CMR also provides quantitative information on cardiac function, perfusion, and vitality, avoiding invasive measurements, radiation, or iodinated contrast exposure. The limitations of this technique are its cost, the difficulties in performing the exam in severely ill patients due to the need for breath-holding, and its scarce availability in all centres.

Among the different CMR sequences, DE-CMR with a long inversion time [97,98], which nulls avascular tissue and identifies LVT as a low-signal intensity intraventricular defect (generally adjacent to an enhanced LV myocardial scarring), was shown to be the most accurate modality for detecting LVT, with a sensitivity of 88% and specificity of 99%. It follows cine-CMR with a sensitivity of 58–79% and specificity of 99% [99,100], which shows LVT next to thinned and dysfunctional LV walls. The evaluation of tissue characterization on top of the tissue morphology improves the detection of LVTs by DE-CMR compared to cine-CMR and contrasted-echocardiography [101]. Anatomic assessment using dark-blood and bright-blood sequences, as well as cardiac motion assessment, using Steady-State Free Precession (SSFP)-based bright-blood images of the beating heart, are also useful for diagnosis.

The CMR, compared to other imaging modalities, has shown its superiority in both the diagnosis and monitoring of the evolution of LVT. In a recent retrospective study of reperfused (post-PCI) STEMI patients who were negative for LV thrombi on TEE (within 24–48 h), CMR viability studies were performed due to either low EF or impaired LV function and 8.8% of the included patients were found to have LVT [101]. Srichai et al. [78] found that in 361 patients with surgically and pathologically confirmed presence or absence of LV thrombus, contrast-enhanced CMR provided the highest sensitivity and specificity (88 ± 9% and 99 ± 2%, respectively) compared with TTE and TEE.

In 194 STEMI patients who underwent primary PCI with stent implantation, CMR was performed at 2–7 days and repeated at 4 months after primary PCI [50]. At baseline, 17 (8.8%) patients had a LV thrombus. At the 4month follow-up, LV thrombus persisted in only 2 of the 17 patients but spontaneously occurred in an additional 12 patients. Another study of 392 STEMI patients showed that 5% of the patients displayed LV thrombus at 1 week, three-quarters of which resolved at 6 months [30]. Moreover, LV thrombus was newly detected in 2% of the total patients at 6 months.

LGE-CMR also revealed that the independent predictors for the formation of LVT in patients with NICM are lower LVEF, larger LV, as well as higher prevalence and larger extension of LGE. A study by Hooks et al. showed that every 5% reduction in LVEF was associated with 36–46% higher odds of LV thrombus, LGE presence was associated with 6-times higher odds of LV thrombus, while every 5% increase in LGE extent was associated with 33% higher odds of LV thrombus [38]. In addition, patients with LV thrombi in NICM have a higher possibility of developing a mobile thrombus instead of an apical thrombus.

A summary of the aforementioned imaging modalities characteristics is shown in Table 1.

## 5. Therapy

The optimal antithrombotic therapy for the treatment and prevention of left ventricular thrombosis (LVT) remains a matter of debate. Although recent studies have shown reduced mortality in individuals with LVT resolution compared to those with persistent thrombus [102], the evidence suggesting that full anticoagulation is more effective at resolving left ventricular thrombus and reducing systemic embolism compared to no or subtherapeutic anticoagulation is still limited [103,104].

In this section, we will cover the pivotal points concerning LVT and medical therapy, namely the choice of antithrombotic agent, the potential role of prophylactic therapy, the optimal therapy duration, and how to start the antithrombotic treatment.

### 5.1. Antithrombotic Agent

With regard to LVT and ischemic heart disease, the first observational studies date back to the last two decades of the 1900s and demonstrated the benefit of anticoagulation with heparin or vitamin K antagonists (VKAs) in terms of both thrombus resolution and lowering embolic complications [105,106,107]. These results have also been confirmed by a subsequent meta-analysis of seven observational studies encompassing a total of 270 patients, which showed an odd ratio of 0.14 (95%CI: 0.04–0.52) with a pooled event rate difference of −0.33 (95%Cl: 0.50–0.16), supporting the hypothesis that systemic anticoagulation was associated with a lower risk of embolic events compared to no anticoagulant therapy [108].

VKAs have always represented the cornerstone of antithrombotic treatment in the course of LVT; indeed, until recently, several guidelines recommended VKAs as the only viable option in this setting [109,110]. One of the first randomized studies in the field was a small controlled trial, which demonstrated that complete thrombus resolution was more frequently achieved in those patients treated with warfarin compared to those receiving either high-dose antiplatelet therapy (aspirin 650 mg daily) or no antithrombotic therapy [111]. Additionally, in individuals treated with warfarin, a longer time in the therapeutic range (TTR) was associated with a lower risk of systemic thromboembolism, according to a recent study (TTR ≥ 50%, 2.9%; TTR < 50%, 19%; *p* = 0.036) [112].

Over the last decade, despite the lack of robust and definite evidence in their favor, the use of DOACs for the treatment of LVT has surged in daily clinical practice. According to a recent retrospective study, the use of direct oral anticoagulants was associated with higher rates of stroke or systemic embolism compared to vitamin K antagonists (HR: 2.64; 95%CI: 1.28–5.43; *p* = 0.01) [113]. In contrast, numerous observational studies [114,115,116,117,118] and small randomized trials [119,120,121,122] highlighted the non-inferiority of DOACs compared to VKAs, especially in terms of clinical outcomes (namely, systemic thromboembolism and bleeding); these findings were also confirmed by a meta-analysis, which did not find any significant difference between the two anticoagulant strategies for thrombus resolution (RR: 1.11 [95%CI: 0.51–2.39]; *p* = 0.76]), bleeding (RR: 1.15 [95%CI: 0.62–2.13]; *p* = 0.57), stroke or systemic embolism (RR: 1.04 [95%CI: 0.64–1.68]; *p* = 0.85), and mortality (RR: 1.09 [95%CI: 0.70–1.70]; *p* = 0.48) [123]. Finally, the observational study by Jones et al. showed a greater and earlier LV thrombus resolution in patients treated with DOACs, compared to those treated with warfarin, at one-year follow-up after the index event (82% vs. 64.4%, respectively; *p* = 0.001). Moreover, the incidence of major bleeding was lower in the DOAC group, compared to the VKA group (0% vs. 6.7%, respectively; *p* = 0.030), with no significant difference in systemic thromboembolic events (5% vs. 2.4%, *p* = 0.388) [124]. These findings were also confirmed by a recent meta-analysis by Gogos et al., encompassing more than 600 patients with post-AMI LV thrombus treated with VKA or DOAC. DOACs showed an approximately 2-fold higher likelihood of thrombus resolution compared to VKAs (OR 1.95 [1.25–3.04]; *p* = 0.003) and a 70% reduction in the risk of systemic embolism (OR 0.30 [0.12–0.75]; *p* = 0.01). Moreover, direct oral anticoagulants were associated with a 54% reduced risk of bleeding than vitamin K antagonists (OR 0.46 [0.26–0.84]; *p* = 0.01), and patients on DOACs had a 63% reduced risk of achieving the composite outcome of safety and efficacy compared to those on VKAs (OR 0.37 [0.23–0.60]; *p* <0.0001) [125].

Based on the aforementioned studies, the latest European Society of Cardiology (ESC) guidelines for the management of acute coronary syndromes introduced DOACs for the treatment of LVT after AMI with the same class of recommendation as VKAs [103], and the same openness toward the use of direct oral anticoagulants in this setting is shared by the American Heart Association (AHA) [104,109].

Left ventricular thrombosis is less common in cardiomyopathies (CM) compared to ischemic heart disease [102]; therefore, there is less evidence on its prevalence and possible complications, as well as on how to treat it once discovered. A retrospective review of 159 individuals with LV thrombus found that 21.5% had a non-ischemic etiology [102]. The average left ventricular (LV) ejection fraction was 32%, and most thrombi were located in the LV apex. Most of the included patients started oral anticoagulation (either VKA or DOAC), and 67% of them received antiplatelet medication. The 62.3% of the study cohort experienced complete LV thrombus reduction within 103 days on average. Patients with poor medical adherence and prothrombotic risk factors, such as active malignancy, inflammatory states, hematological disorders, or chronic kidney disease, were more likely to experience recurrence or an increase in the size of the LV thrombus. In conclusion, considering the paucity of available evidence, mostly based on retrospective registry data and small prospective observational studies [126,127,128], major scientific societies are unable to provide definite indications regarding the optimal antithrombotic strategy (namely, which oral anticoagulant is the preferred choice) for patients with left ventricular thrombus and underlying cardiomyopathy [104,129,130,131].

Table 2 shows an overview of the main studies evaluating the antithrombotic strategies for LVT treatment. 

### 5.2. Antithrombotic Preventive Therapy

Although the advent of modern percutaneous coronary reperfusion techniques has significantly reduced the prevalence of LVT following AMI, its incidence, particularly in patients with anterior infarction, is still not negligible, with peaks as high as 26% according to some studies [5,35,132,133]; furthermore, the presence of LVT significantly worsens the prognosis of the affected patients [134].

Previous guidelines suggested considering the administration of VKAs in patients with STEMI who are considered at high risk for LVT (namely, those with anterior infarction and apical akinesis/dyskinesis), even without demonstrable left ventricular mural thrombus on echocardiography or other imaging modalities [109,110]. In this setting, a lower INR goal (2–2.5) for those receiving concomitant dual antiplatelet therapy (DAPT) was also suggested [109].

However, over the years, the prognostic relevance of major bleeding after AMI has been largely acknowledged; thus, the extension of full anticoagulation beyond the acute phase of AMI should be carefully considered and undertaken only in cases of clear indication (mechanical heart valves, concomitant venous thromboembolism, AF/AFL, or well-established diagnosis of LVT) [103,135,136,137,138]. Hence, more recent recommendations from the ESC discourage the routine administration of post-procedural anticoagulation in AMI after primary percutaneous coronary intervention (PCI) [103,139,140].

A recent controlled study enrolled almost 300 patients with anterior STEMI who had undergone primary PCI. Patients were randomized to receive either low-dose rivaroxaban (2.5 mg twice daily for 30 days) and DAPT or only DAPT. The subjects in the rivaroxaban arm presented a lower rate of LVT formation (0.7% vs. 8.6%; HR = 0.08 [95%CI: 0.01–0.62]; *p* = 0.015) and net adverse clinical events (6.5% vs. 16.4%; HR = 0.37 [95%CI: 0.17–0.80]; *p* = 0.011), without increased bleeding (3.6% vs. 1.7%; HR = 2.08 [95%CI: 0.38–11.33]; *p* = 0.399) [141]. Based on these results, the American Heart Association suggests an individual-based evaluation by balancing each patient’s thrombotic and hemorrhagic risk factors before starting any prophylactic anticoagulation therapy in this clinical setting [104].

Regarding cardiomyopathies, the thromboembolic risk differs across phenotypes, with cardiac amyloidosis, hypertrophic cardiomyopathy (HCM), restrictive cardiomyopathy (RCM), and left ventricular non-compaction cardiomyopathy (LVNC) being associated with a particularly increased risk of stroke. Moreover, individuals with cardiomyopathies have a higher prevalence of AF compared to the general population, with figures up to 30%, representing a further thromboembolic risk factor in addition to their intrinsic thrombogenic risk [142,143,144,145]. A registry encompassing more than 3000 patients evaluating thromboembolic events in AF vs. non-AF individuals with underlying cardiomyopathies showed worse outcomes for the population with cardiomyopathy and concomitant AF, with an annual incidence of stroke and transitory ischemic attack (TIA) around three times greater [146]. Therefore, major scientific societies have remarked on the role of prophylactic oral anticoagulation in patients with cardiac amyloidosis, HCM, or RCM and concomitant atrial fibrillation (AF) or atrial flutter (AFL), regardless of the CHA_2_DS_2_-VASc score [129,147]. Additionally, on the basis of a small retrospective study [148], the American Heart Association suggests considering empiric anticoagulant therapy in patients with cardiac amyloidosis and sinus rhythm. This recommendation particularly applies when decreased A-wave amplitude and left atrial appendage velocities are observed on echocardiography, attesting to the typical atrial dysfunction seen in amyloid cardiomyopathy [147,148]. No specific indication has been provided by major scientific societies concerning preventive anticoagulant therapy in individuals affected by LVNC; nonetheless, according to a systematic review and some observational studies, no clear benefit is derived from preventive anticoagulant therapy unless there are other concomitant thromboembolic risk factors (i.e., AF/AFL and/or LV ejection fraction < 40%) [128,149,150].

In contrast, prophylactic anticoagulation in patients with AF/AFL and dilated cardiomyopathy (DCM), non-dilated left ventricular cardiomyopathy (NDLVC), or arrhythmogenic right ventricular cardiomyopathy (ARVC) follows the same indications as for the general population, namely, on an individual basis, considering the CHA_2_DS_2_-VASc score [129].

**Figure 3:**
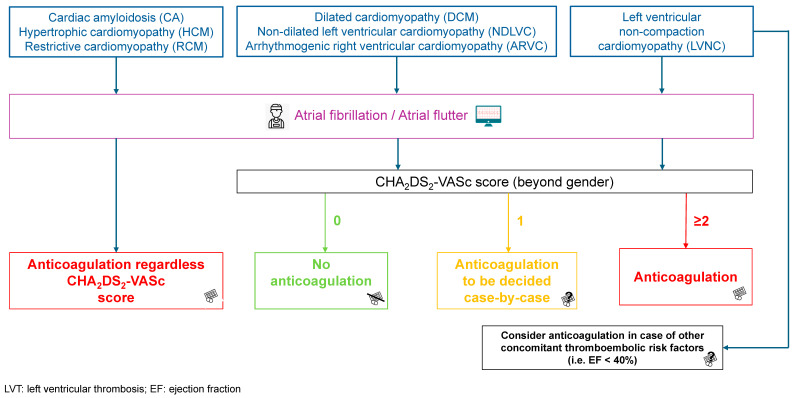
Evidence-based flowchart for the management of antithrombotic preventive therapy in non-ischemic cardiomyopathy-related LVT.

Figure 3 shows an evidence-based flowchart for the management of antithrombotic preventive therapy in non-ischemic cardiomyopathy-related LVT.

To date, no randomized study has compared VKAs and DOACs in individuals with cardiomyopathy has been conducted; thus, no specific indication concerning the choice of the anticoagulant strategy is available [129]. However, data suggest that direct oral anticoagulants in this setting could be safely used in this setting in a manner similar to that used in the general population [151,152,153,154,155,156].

Table 3 shows an overview of the main studies evaluating the antithrombotic strategies for LVT prevention.

### 5.3. Duration of Anticoagulant Therapy

The optimal duration of anticoagulant therapy for LVT following myocardial infarction remains unclear. Indeed, no randomized controlled trial has been designed to establish significant differences across duration regimens. Moreover, following acute MI, anticoagulation is added on top of single or dual antiplatelet therapy, thus representing a considerable additional bleeding risk factor.

In the absence of absolute contraindications, in the case of LVT detection, immediate initiation of full-dose anticoagulation is mandatory, and it should be continued for at least 3–6 months after the thrombus diagnosis [103,104,110], as observed in most of the available randomized studies on the topic [119,120,121,122].

Cardiac imaging plays a pivotal role in establishing the actual duration of anticoagulant therapy for LVT after AMI. Periodic assessment of thrombus clearance through transthoracic echocardiography (less frequently, contrast echocardiography or cardiac MRI) is mandatory, as stated by all major scientific societies [103,104,158]. Indeed, the serial evaluation of the thrombotic burden aiming at verifying complete thrombus resolution is crucial to establish the continuation of the anticoagulant therapy, which should be prolonged according to the findings of the follow-up investigation [103]. This evidence explains why, in most real-world studies, the duration of anticoagulation largely exceeds the period suggested by current guidelines [102,113,115].

If cardiac imaging proving thrombus clearance is performed for other clinical reasons before the third month from the diagnosis, the interruption of anticoagulation should be anyway avoided in case of persisting abnormalities in cardiac wall motion (i.e., akinesis/dyskinesis) [104]: indeed, has already been acknowledged that the risk of thrombotic recurrence is the highest during the first trimester after an acute MI [159]. However, if the thrombus has cleared and the left ventricular function and wall motion abnormalities have improved, a shorter regimen could be considered [104].

Regarding thrombus characteristics, a thicker thrombus and mural thrombus predict the risk of LV thrombus persistence/recurrence after 3 months of oral anticoagulation [160], while a partial or highly mobile thrombus, defined so on its visual assessment, is associated with the early thrombus resolution [80].

The duration of anticoagulation therapy for LV thrombus and CM remains an open question. Similar to what happens with ischemic heart disease, a period of full anticoagulant therapy of 3–6 months appears to be reasonable, based on the results of small observational studies [126,127,128]. Nevertheless, there are clinical scenarios in which prolonged or indefinite anticoagulant therapy is advisable, namely in the presence of a strong thrombogenic drive, either local (lack of improvement in LV systolic function despite the best medical therapy) or systemic (inflammation or hypercoagulable states, such as malignancies or previous thromboembolic events) [104]. In this scenario, a comprehensive case-by-case evaluation of hemorrhagic risk assessment is mandatory, and patient preferences should be taken into consideration in the decision-making process [104]. In the absence of absolute contraindications, as mentioned above, indefinite anticoagulation should be initiated in cases of concomitant AF/AFL [147,161].

Table 4 shows an overview of the main studies evaluating the duration of anticoagulation for LVT treatment.

### 5.4. Which Antithrombotic Treatment to Start with

Currently, there is no solid evidence concerning how to start anticoagulation in patients diagnosed with LVT, regardless of the underlying cause. In the VKA era, bridging therapy with parental anticoagulation was a regular part of LVT treatment. However, more recently, because of the widespread use of direct oral anticoagulants, the single-drug approach with DOACs has also been used in this clinical setting.

It is self-evident that initial parental anticoagulation still represents the preferred treatment strategy in all patients with LVT (regardless of the underlying cause) and hemodynamic instability. Moreover, heparin lead-in therapy should also be considered for high bleeding risk patients or, in the case of ACS, if heart surgery is planned; in these cases, the use of drugs with a shorter half-life and a reversal agent available (i.e., protamine sulfate for unfractionated heparin) represents the strategy of choice. In the remaining cases, there does not seem to be any contraindication to starting anticoagulation with a DOAC; current guidelines do not specifically address this issue, merely suggesting the use of oral anticoagulation with VKA or DOAC [103].

To the best of our knowledge, the use of a short-period loading dose of DOAC in LVT treatment has never been investigated, even for those agents for which this strategy has been approved for other indications (i.e., apixaban and rivaroxaban for venous thromboembolism). Finally, although in some studies considering concomitant DAPT the low-dose DOAC has been the default strategy [162], most current and ongoing studies have adopted the same DOACs dose reduction criteria as for atrial fibrillation [163,164,165].

### 5.5. Future Perspectives

Several clinical trials are currently investigating different antithrombotic strategies for the medical treatment and prevention of left ventricular thrombosis. The ACTonLVT study is a randomized, parallel assignment, open-label clinical trial including patients with LVT after STEMI, randomized to receive DAPT (the choice of P2Y12 inhibitor is at the discretion of the clinician) vs. DAPT plus rivaroxaban (15 mg daily) [163]. The EARLYmyo-LVT [162] and WaRMIN [164] studies fit into the same clinical scenario: STEMI patients with LVT in treatment with DAPT were assigned to warfarin (INR goal: 2–2.5 and 2–3, respectively) vs. rivaroxaban (15 mg daily and 20 mg daily, according to label, respectively). The investigator-initiated, open-label, randomized and multicenter RESOLVE-AMI clinical trial compares apixaban (5 mg or 2.5 mg twice daily, according to label) and warfarin (INR goal: 2–3) in patients diagnosed with LVT after AMI. Finally, the WRAP study was a randomized, parallel assignment, open-label, three-arm study comparing the use of warfarin (INR goal: 2–3), rivaroxaban (20 mg daily), and apixaban (5 mg or 2.5 mg twice daily) in hemodynamically stable patients with LVT from any cause [165].

In contrast, concerning LVT preventive therapy, the prospective, randomized, open-label, blinded end-point APERITIF study randomized patients with anterior STEMI to receive DAPT (aspirin and clopidogrel or ticagrelor) vs. DAPT plus low-dose DOAC (rivaroxaban 2.5 mg daily) [166].

## 6. Conclusions

Despite continuous improvements in the management of acute myocardial infarction and cardiomyopathies, left ventricular thrombosis still represents a frightening complication of both clinical scenarios, weighing on the affected patients with high morbidity and mortality rates. Prompt diagnosis, swift medical therapy initiation, and serial imaging are mandatory to improve patient outcomes. Despite conclusive evidence in their favor, DOACs are becoming of common use in this setting; nonetheless, the results of numerous ongoing clinical trials are eagerly awaited in order to establish the optimal antithrombotic strategy for patients with left ventricular thrombosis.

## Figures and Tables

**Table 1:** Key characteristics of different imaging modalities available for LVT diagnosis and monitoring.

	Pros	Cons	Findings	Sensitivity	Specificity
TTE	Easily available, cheap, safe	Impaired acoustic window, operator depending, difficult detection of mural and small thrombi	Echo-dense mass with well-defined margins, separated from the endocardium	21–35% With IV echo contrast: 61–64% With apical wall motion scoring: 100%	86–88%
TEE	Cheap, safe	Cardiac apex not always assessable	Echo-dense mass with well-defined margins, separated from the endocardium	58%	98%
CCT	High precision	Limited tissue characterization of the masses, contrast-based exam	Cavity-filling defect	91% With spectral tomography dual-substance separation: 94%	97%
Contrast ventriculography	High specificity	Low spatial resolution, invasive, contrast-based exam, acute phase exam, not repeatable	Cavity-filling defect	30%	85–90%
CMR	High spatial and temporal resolution, high soft-tissue contrast	Costly, time consuming, difficult to perform in acutely ill patients	Low-signal intensity intraventricular defect	DE-CMR with long inversion time: 88% Cine-CMR: 58–79%	DE-CMR with long inversion time: 99% Cine-CMR: 99%

LVT = left ventricular thrombosis; TTE = transthoracic echocardiography; IV = intravenous; TEE = transesophageal echocardiography; CCT = contrast-enhanced cardiac computed tomography; CMR = cardiac magnetic resonance.

**Table 2:** Key studies evaluating antithrombotic strategies for LVT treatment.

Reference	Study Design	Patients (n)	Outcomes	Key Results	Limitations
Maniwa N. et al. (2018) [112]	Retrospective cohort study	84	Warfarin with TTR ≥ 50% vs. warfarin with TTR < 50% in patients with first MI and LVT. Evaluation of systemic embolism.	Longer TTR was associated with a lower risk of systemic embolism (TTR ≥ 50%: 2.9% vs. TTR < 50%: 19%; *p* = 0.036).	Small sample size. Retrospective design.
Robinson A.A. et al. (2020) [113]	Retrospective cohort study	514	DOACs vs. warfarin in patients with LVT. Evaluation of stroke and embolic events.	DOACs were associated with a higher risk of stroke and systemic embolism (HR: 2.64 [95%CI: 1.28–5.43]; *p* = 0.01).	No safety outcomes. Retrospective design.
Daher J. et al. (2020) [114]	Retrospective cohort study	59	VKAs vs. DOACs in patients with LVT. Evaluation of thrombus resolution and embolic events.	No difference was found for thrombus resolution (VKAs: 71.4%; DOACs: 70.6%; *p* = 0.9) and embolic events (VKAs: 9.5%; DOACs: 11.8%; *p* = 0.8). Patients without thrombus resolution on DOAC were switched to VKA (INR: 3–4) with subsequent complete thrombus clearance.	Small sample size. No safety outcomes. Retrospective design.
Iqbal H. et al. (2020) [115]	Retrospective cohort study	84	VKAs vs. DOACs in patients with LVT. Evaluation of thrombus resolution, embolic events, bleeding, and all-cause death.	No difference was found for thrombus resolution (VKAs: 76%; DOACs: 65%; *p* = 0.33), stroke (VKAs: 2%; DOACs: 0%; *p* = 0.55), bleedings (VKAs: 10%; DOACs: 0%; *p* = 0.13), and all-cause death (VKAs: 10%; DOACs: 14%; *p* = 0.61).	Small sample size. No standardized follow-up imaging. Retrospective design.
Kouvaras G. et al. (1990) [111]	RCT	60	Warfarin vs. aspirin (650 mg daily) vs. placebo in patients with MI and LVT. Evaluation of thrombus resolution, embolic events, and bleeding.	Thrombus resolution was observed in 60% of the patients in the warfarin group, 45% in the aspirin group and 10% in the placebo group. Three patients in the placebo group had an embolic event. Three patients in the warfarin group had a bleeding event.	Small sample size. Diagnostic limitations due to old echocardiographic technology. No bleeding definition.
Abdelnabi M. et al. (2021) [122]	RCT	79	Warfarin vs. rivaroxaban (20 mg daily) in patients with LVT. Evaluation of thrombus resolution, stroke, systemic embolism, and major bleeding (ISTH criteria).	No difference was found for thrombus resolution (warfarin: 80%; rivaroxaban: 87.2%; *p* = 0.39), stroke (warfarin: 10%; rivaroxaban: 0%; *p* = 0.08), systemic embolism (warfarin: 5%; rivaroxaban: 0%; *p* = 0.25), and major bleedings (warfarin: 15%; rivaroxaban: 5.1%; *p* = 0.11).	Small sample size. Unblinded.
Alcalai R. et al. (2022) [120]	RCT	35	Warfarin vs. apixaban (5 mg daily, according to label) in patients with MI and LVT. Evaluation of thrombus resolution, major bleeding (ISTH criteria), and stroke/systemic embolism.	No difference was found for thrombus resolution (warfarin: 93%; apixaban: 94%; *p* = 1). Due to the low event rate, the study was underpowered to assess significant differences for major bleedings (warfarin: 13.3%; apixaban: 0%; *p* = N/A) and stroke/systemic embolism (warfarin: 6.6%; apixaban: 0%; *p* = N/A).	Small sample size. Unblinded. Underpowered for key outcomes.
Youssef A.A. et al. (2022) [121]	RCT	50	Warfarin vs. apixaban (5 mg daily) in patients with anterior MI and LVT. Evaluation of thrombus resolution and major adverse cardiovascular events: all-cause death/ischemic stroke or TIA/MI or acute peripheral artery emboli/clinically relevant bleedings (BARC criteria).	No difference was found for thrombus resolution (warfarin: 80%; apixaban: 76%; *p* = 0.3). No major adverse cardiovascular event was reported.	Small sample size. Unblinded.
Mansouri P. et al. (2024) [119]	RCT	52	Warfarin vs. rivaroxaban (20 mg daily) on top of DAPT (aspirin + clopidogrel) in patients with MI undergoing PCI and LVT. Evaluation of thrombus resolution and bleeding.	No difference was found for thrombus resolution (warfarin: 69.2%; rivaroxaban: 76.9%; *p* = 0.53) and bleeding (warfarin: 1%; rivaroxaban: 1%; *p* = 1).	Small sample size. Single-center. Unblinded. No bleeding definition.
Vaitkus P.T. et al. (1993) [108]	Meta-analysis	270	Warfarin vs. placebo in patients with anterior MI and LVT.	Anticoagulation with warfarin reduced the embolic risk compared to no anticoagulation (OR 0.14 [95%CI: 0.04–0.52]).	Small sample size of the included studies. Diagnostic limitations due to old echocardiographic technologies. No safety outcomes.
Gogos C. et al. (2024) [125]	Meta-analysis	605	VKAs vs. DOACs in patients with MI with evidence of LVT. Evaluation of thrombus resolution, systemic embolism, and bleeding.	DOACs were associated with higher rates of thrombus resolution (OR 1.95 [95%CI: 1.25–3.04]; *p* = 0.003) and lower risk of systemic embolism (OR 0.30 [95%CI: 0.12–0.75]; *p* = 0.01) and bleedings (OR 0.46 [95%CI: 0.26–0.84]; *p* = 0.01), compared to VKAs.	Observational and randomized studies pooled together. Subgroup analyses are prone to confounding due to the small sample of the included studies. No univocal definition of bleeding across the included studies.

TTR: time to range; MI: myocardial infarction; LVT: left ventricular thrombosis; DOACs: direct oral anticoagulants; HR: hazard ratio; CI: confidence interval; VKAs: vitamin K antagonists; INR: international normalized ratio; ISTH: Internation Society on Thrombosis and Haemostasis; N/A: not available; TIA: transient ischemic attack; DAPT: dual antiplatelet therapy; PCI: percutaneous coronary intervention; RCT: randomized controlled trial; OR: odds ratio.

**Table 3:** Key studies evaluating antithrombotic strategies for LVT prevention.

Reference	Study Design	Patients (n)	Outcomes	Key Results	Limitations
Le May M.R. et al. (2015) [135]	Retrospective cohort study	460	Warfarin vs. no anticoagulation, on top of DAPT (aspirin + clopidogrel) in patients with anterior MI undergoing PCI. Evaluation of NACE (all-cause death, stroke, reinfarction, and major bleeding).	Patients treated with warfarin had a higher rate of NACE (14.7% vs. 4.6%; *p* = 0.001). Rates of death (5.4% vs. 1.5%; *p* = 0.04) and stroke were also higher (3.1% vs. 0.3%; *p* = 0.02) in the warfarin group.	No data on INR at the time of bleeding. Retrospective design.
Shavadia J.S. et al. (2017) [136]	Retrospective cohort study	2032	Warfarin vs. no anticoagulation in patients with MI. Evaluation of composite of stroke/TIA/systemic embolism/all-cause death and bleeding requiring hospitalization in high-risk STEMI.	No difference was found for the composite outcome (warfarin: 23.3% vs. no anticoagulation: 25.3%; OR: 0.96 [95%CI: 0.60–1.55]) and bleedings (warfarin: 2.5% vs. no anticoagulation: 1.2; OR: 2.17 [95%CI: 0.43–10–96]).	Unable to exclude patients developing AF during the observation period. Retrospective design.
El-Am E.A. et al. (2019) [148]	Retrospective cohort study	172	Patients with atrial arrhythmias and cardiac amyloidosis vs. the control group with atrial arrhythmias and no cardiac amyloidosis. Evaluation of direct current cardioversion cancellation rate due to intracardiac thrombus at TEE.	Patients with cardiac amyloidosis had higher rates of intracardiac thrombus compared to the control group (cardiac amyloidosis: 81%; control group: 25%; *p* = 0.02).	Small sample size. Retrospective design.
Peters F. et al. (2014) [157]	Prospective cohort study	55	Warfarin vs. no anticoagulation in patients with LVNC.	1 out of 16 patients had a systemic thromboembolic event in the warfarin group. Non-major or minor bleeding occurred in the warfarin group.	Small sample size. No bleeding definition.
Zhang Z. et al. (2022) [141]	RCT	279	Low-dose rivaroxaban (2.5 mg twice daily) vs. no anticoagulation, on top of DAPT (aspirin + clopidogrel/ticagrelor) in patients with anterior MI undergoing PCI. Evaluation of LVT formation (within 30 days), NACE (all-cause death, LVT, systemic embolism, rehospitalization, and bleedings), and bleedings (ISTH criteria).	Patients treated with rivaroxaban showed a lower rate of LVT formation (rivaroxaban: 0.7% vs. no anticoagulation: 8.6%; HR 0.08 [95%CI: 0.01–0.62]) and NACE (rivaroxaban: 6.5% vs. no anticoagulation: 16.4%; HR: 0.37 [95%CI: 0.17–0.80]), without increase in bleedings (rivaroxaban 3.6% vs. no anticoagulation: 1.7%; HR: 2.08 [95%CI: 0.38–11.33]).	Small sample size. High dropout rate. Short follow-up.

DAPT: dual antiplatelet therapy; MI: myocardial infarction; PCI: percutaneous coronary intervention; NACE: net adverse clinical events; INR: international normalized ratio; TIA: transitory ischemic attack; STEMI: ST-elevation myocardial infarction; OR: odds ratio; CI: confidence interval; AF: atrial fibrillation; TEE: transesophageal echocardiography; LVNC: left ventricular non-compaction cardiomyopathy; RCT: randomized controlled trial; LVT: left ventricular thrombosis; ISTH: Internation Society on Thrombosis and Haemostasis; HR: hazard ratio.

**Table 4:** Key studies evaluating the duration of anticoagulation for LVT treatment.

Reference	Study Design	Patients (n)	Duration of Anticoagulation, Days	Limitations
Lattuca B, et al. (2020) [102]	Retrospective cohort study	159	508 (15–986) *	Diagnosis and follow-up with TTE. Retrospective design.
Robinson A. A., et al. (2020) [113]	Retrospective cohort study	514	All anticoagulants: 207 (57–491.4) * DOACs: 95.5 (69–373) * Warfarin: 241 (47–579.5) *	Diagnosis and follow-up with TTE. Retrospective design.
Iqbal H. et al. (2020) [115]	Retrospective cohort study	84	All anticoagulants: 677 ± 568 ° DOACs: 545 ± 368 ° Warfarin: 724 ± 619 °	Small sample size. Diagnosis and follow-up with TTE. Retrospective design.
Abdelnabi M, et al. (2021) [122]	RCT	79	180	Small sample size. Unblinded.
Alcalai R. et al. (2022) [120]	RCT	35	89 (85–94) *	Small sample size. Unblinded. Underpowered for key outcomes.
Youssef A.A. et al. (2022) [121]	RCT	50	180	Small sample size. Unblinded.
Mansouri P. et al. (2024) [119]	RCT	52	90 (average)	Small sample size. Single-center. Unblinded. No bleeding definition.

* median, IQR (interquartile range); ° mean, min–max. TTE: transthoracic echocardiography; DOACs: direct oral anticoagulants; RCT: randomized controlled trial.
